# Postoperative Thrombocytopenia in Cardiac Surgery: Patterns, Differential Diagnosis and Management of Heparin-Induced Thrombocytopenia (HIT)

**DOI:** 10.3390/jcm14248765

**Published:** 2025-12-11

**Authors:** Fotini Ampatzidou, Eleni Argyriadou, Catherine Amaniti, Despoina Sarridou

**Affiliations:** 1 ICU Department, George Papanikolaou General Hospital of Thessaloniki, 570 10 Exochi, Greece; 2 Department of Anesthesiology and Intensive Care, AHEPA University Hospital, 546 36 Thessaloniki, Greece; ahepa-hos@med.auth.gr (E.A.); aamaniti@auth.gr (C.A.); dodasarri@yahoo.gr (D.S.); 3 Faculty of Health Sciences, School of Medicine, Aristotle University of Thessaloniki, 541 24 Thessaloniki, Greece

**Keywords:** thrombocytopenia, cardiac surgery, heparin-induced thrombocytopenia

## Abstract

**Background**: Postoperative thrombocytopenia is a frequent finding after cardiac surgery, affecting approximately 10–40% of patients. The early decline in platelet count, typically observed within the first 48–72 h, is usually explained by hemodilution, perioperative platelet consumption, and the inflammatory or mechanical impact of cardiopulmonary bypass (CPB). However, a secondary decrease occurring beyond postoperative day 5 should raise suspicion for more serious causes, the most important being heparin-induced thrombocytopenia (HIT), a rare but potentially life-threatening complication. **Objectives**: This review aims to outline the characteristic patterns of postoperative thrombocytopenia in cardiac surgery, provide a practical framework for differential diagnosis, and summarize up-to-date evidence on the diagnosis and management of HIT. **Methods**: A narrative review was conducted through searches in PubMed, Scopus, and Embase up to September 2025. **Conclusions**: Recognizing platelet count trends early and applying a stepwise diagnostic approach can help distinguish benign postoperative thrombocytopenia from HIT and other critical causes. The combination of clinical assessment using the 4Ts score and appropriate confirmatory testing remains central to accurate diagnosis. Tailoring anticoagulant therapy to individual patient requirements—including surgical procedure, renal and hepatic function, and bleeding risk—is essential for reducing thrombotic events and improving postoperative outcomes.

## 1. Introduction

Thrombocytopenia is defined as a pathological decrease in the number of platelets below <150,000/μL. Thrombocytopenia is classified according to its severity, and the classification is mainly based on the platelet count and the risk of bleeding (Table 1). The risk of bleeding in thrombocytopenia is not determined by platelet count alone; the relationship is not linear and depends on platelet function as well as patient-specific variables such as comorbidities, medications, and clinical conditions. Elderly patients are generally at higher risk due to vascular fragility and associated conditions, while children may tolerate lower platelet counts better [1]. The underlying cause of thrombocytopenia is another critical factor.

According to a broader definition, thrombocytopenia is a relative reduction in platelet count, approximately 30–50% decrease from the baseline value, even if the absolute count remains within the normal reference range [2].

Platelets or thrombocytes are small, anucleate cell fragments derived from megakaryocytes in the bone marrow and have a life span of 7–10 days. The body strives to maintain a stable platelet count. This process is regulated by the secretion of thrombopoietin (TPO), a hormone produced primarily by the liver and kidneys. Thrombopoietin acts on megakaryocytes, stimulating their proliferation and differentiation into platelets [3].

Both the number and functionality of platelets are prerequisites for successful hemostasis and thrombus formation. Platelets also appear to play a role in the wound healing process by releasing growth factors. Additionally, they contribute to the immune response through interactions with pathogens and by secreting immune modulators that are chemotactic for neutrophils, monocytes, and lymphocytes. These interactions result in the formation of platelet–granulocyte or platelet–leukocyte aggregates, which further trigger inflammatory responses [4]. The aim of this narrative review is to provide a clinically focused synthesis of postoperative thrombocytopenia in cardiac surgery, with particular emphasis on the identification, differential diagnosis, and evidence-based management of heparin-induced thrombocytopenia (HIT). By integrating current pathophysiological insights with practical diagnostic tools, this review seeks to assist clinicians in distinguishing expected postoperative platelet trends from pathological patterns requiring urgent evaluation and intervention.

## 2. Methods

A narrative review was conducted through searches in PubMed, Scopus, and Embase up to September 2025. The search strategy included terms related to thrombocytopenia, cardiac surgery, cardiopulmonary bypass, and heparin-induced thrombocytopenia. Relevant clinical studies, systematic reviews, and consensus guidelines were reviewed and analyzed.

### 2.1. Postoperative Thrombocytopenia

Postoperative thrombocytopenia occurs in approximately 30–60% of cases due to perioperative interventions, blood transfusions, and the administration of anticoagulant drugs (such as heparin). In cases of complications leading to prolonged ICU stay, the likelihood of sepsis and multiple organ failure increases, consequently raising the risk of thrombocytopenia [5]. In perioperative bleeding, as well as in trauma, thrombocytopenia may result from consumption coagulopathy and dilution caused by aggressive fluid resuscitation. This typically occurs within the first 4 days postoperatively, while platelet counts usually return to their previous levels approximately 2 weeks after surgery. In traumatic cases with critical injuries, the admission platelet count is usually normal, but a rapid decrease in platelet count occurs within hours after hospitalization. In medical patients, the dynamics of platelet count changes depend largely on the underlying condition, such as sepsis [6].

### 2.2. Thrombocytopenia in Critical Care Patients

Postoperative thrombocytopenia that presents on Postoperative Day 5 (POD5) or later is classified as late-onset thrombocytopenia. In such cases, the diagnostic approach should follow the principles applied to thrombocytopenia in critically ill patients, as the differential diagnosis is broad and may include potentially life-threatening conditions.

In the PLOT-ICU cohort study, which included 1168 patients across 52 ICUs and excluded postoperative patients, the overall incidence of thrombocytopenia was 43.2%. Baseline thrombocytopenia was present in 23.4% of patients (95% CI: 20.0–26.0), while 19.8% (95% CI: 17.6–22.2) developed thrombocytopenia during their ICU stay. It is important to note that elective surgical cases were not included in this study [7].

Sepsis is a serious and often life-altering complication for patients undergoing cardiothoracic surgery. It arises from a dysregulated inflammatory response to infection, which can trigger significant organ dysfunction and increase mortality. Despite advances in perioperative care, postoperative sepsis remains a common event in cardiothoracic procedures and continues to be strongly associated with poorer clinical outcomes [8,9].

Sepsis triggers a “storm” of immune and coagulation responses, which collectively reduce platelet numbers and impair their function [10]. Decreased platelet production in sepsis is the result of a combined effect of bone marrow suppression by cytokines and toxins, nutritional deficiencies that affects bone marrow function and finally reduced thrombopoietin production [11].

However, bibliographic data show that in cases of severe endotoxemia, the marked increase in pro-inflammatory markers—such as TNF-α, IL-1, IL-6, and IL-8—may actually act as thrombopoietic agents, meaning they can stimulate megakaryopoiesis [12]. This is why in some septic patients, we can see a reactive thrombocytosis (increase in platelets) instead of thrombocytopenia.

Immune-Mediated Platelet Destruction is another mechanism in sepsis. Up to 30% of septic patients have platelet autoantibodies contributing to low counts [13]. Activated macrophages engulf and destroy platelets because of the hemophagocytosis in septic patients [14].

In addition, sepsis causes widespread endothelial injury, exposing pro-thrombotic surfaces. Platelets adhere excessively and are consumed in microthrombi leading to consumptive thrombocytopenia. This overlaps with disseminated intravascular coagulation (DIC), a dangerous and potentially fatal complication of sepsis [15]. Moreover, sepsis not only reduces platelet count but also causes platelet dysfunction, with impaired aggregation and reduced hemostatic capacity leading in weaker clot formation [16].

It is also important to keep in mind pseudothrombocytopenia, a laboratory artifact rather than a true reduction in platelet count. This phenomenon is usually related to platelet clumping in EDTA-anticoagulated blood samples, and it may also be seen in patients receiving GPIIb/IIIa receptor antagonists [17,18].

### 2.3. Cardiac Surgery and Thrombocytopenia

**Figure 1:**
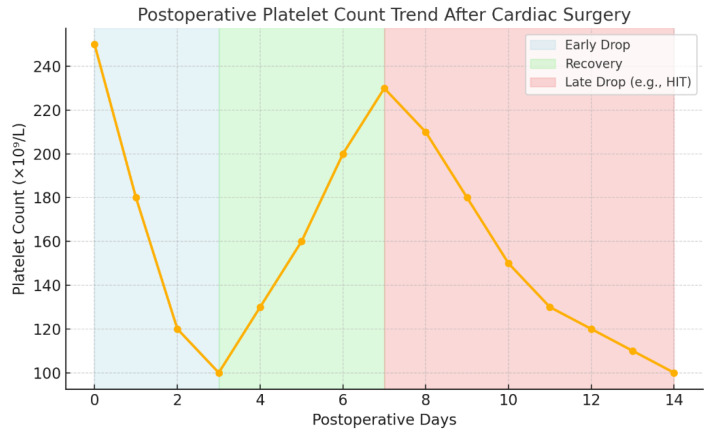
Illustrative postoperative platelet count trend following cardiac surgery.

Postoperative thrombocytopenia occurs in about 10–40% of patients, with the exact incidence varying according to the definition criteria applied [19]. The platelet count typically reaches its lowest point 48 to 72 h after surgery, showing an average reduction of about 50% from baseline levels [20]. In cardiac surgery, particularly with the use of cardiopulmonary bypass (CPB), usually there is a typical pattern of postoperative thrombocytopenia: an early platelet fall within the first 48–72 h due to hemodilution, consumption, and cardiopulmonary bypass–related mechanical effects; a recovery phase between postoperative days 3 and 7 reflecting normalization of platelet production; and a late secondary decline beyond day 7, which may indicate pathological causes such as heparin-induced thrombocytopenia (HIT) [Figure 1]. One proposed mechanism involves blood–membrane interactions and shear stress within the extracorporeal circuit, leading to platelet activation and degranulation [21,22].

Hemodilution is a well-recognized cause of thrombocytopenia in critically ill patients, particularly in the ICU and perioperative settings as well as trauma patients. It usually occurs after massive fluid resuscitation or massive transfusion (red blood cells, crystalloids, colloids) without proportionate platelet replacement. The mechanism is a dilutional decrease in circulating platelet concentration, rather than increased destruction or reduced production. Thrombocytopenia in this context is typically transient and often accompanied by dilution of coagulation factors, which may worsen bleeding risk.

Additionally, an inflammatory response induced by CPB has been suggested as a major contributing factor. The activation of inflammatory cytokines such as TNF-α, IL-8, IL-10, IL-6, and IL-1β may promote systemic autoimmune-mediated platelet clearance [23]. Furthermore, studies have demonstrated significantly greater platelet activation after on-pump compared with off-pump coronary artery bypass grafting (CABG), supporting the role of CPB-related mechanical and inflammatory mechanisms in postoperative platelet dysfunction [24]. Certain cardiac surgical procedures, particularly aortic valve replacement (AVR), have been strongly associated with postoperative thrombocytopenia. This phenomenon is observed more frequently following the use of bioprosthetic valves compared with mechanical prostheses, and has also been reported with more modern techniques such as rapid-deployment, new sutureless valves and transcatheter aortic valve bioprostheses (TAVR) [25]. Evidence suggests that some bioprosthetic valve models are more prone to platelet reduction, possibly due to differences in surface materials, coating processes, and blood–valve interface characteristics. Furthermore, the duration of CPB has been identified as a critical determinant in the onset and severity of postoperative thrombocytopenia, highlighting the multifactorial and procedure-specific nature of this condition in valvular heart surgery. Moreover, chemical agents used for pericardial tissue stabilization during the manufacturing of bioprosthetic leaflets may further affect platelet function. Previous studies have demonstrated that bioprostheses stored in glutaraldehyde-based solutions are associated with a lower incidence of thrombocytopenia compared with those preserved in homocysteic acid–based solutions, suggesting that the preservation medium may influence postoperative platelet dynamics. Altogether, these findings highlight the multifactorial and procedure-specific nature of thrombocytopenia following valvular heart surgery [26,27,28]. Ρostoperative thrombocytopenia has been correlated with a higher risk of adverse clinical outcomes, including acute kidney injury (AKI), stroke, and increased mortality, highlighting its potential prognostic significance in valvular heart surgery [29].

Particular attention should be given to the mechanical destruction of platelets observed in patients supported with extracorporeal devices. Several commonly used modalities are associated with this phenomenon: Intra-aortic balloon pump (IABP), Impella device, Hemodialysis and the use of Εxtra Corporeal Membrane Oxygenator (ΕCMO).

The use of intra-aortic balloon pump has been associated with active platelet destruction and consumption, due to repeated balloon inflation and deflation within the aorta [30]. Impella device is a percutaneous mechanical circulatory support system primarily used in the management of cardiogenic shock and acute myocardial ischemia. While it provides significant hemodynamic stabilization, its use is associated with specific complications, most notably thrombocytopenia. Thrombocytopenia often develops within the first 24 h after device implantation and is mainly driven by two pathophysiological mechanisms: Increased shear stress, leading to platelet activation and destruction and platelet adhesion and deposition on the device surface, resulting in platelet consumption [31]. The clinical significance of thrombocytopenia lies in both the increased risk of bleeding and the need to differentiate it from other causes, such as heparin-induced thrombocytopenia (HIT), which may also occur in patients receiving anticoagulation during this type of support.

### 2.4. Heparin-Induced Thrombocytopenia

Hemorrhagic complications are a common occurrence during heparin therapy; however, the most frequent non-hemorrhagic complication is Heparin-Induced Thrombocytopenia (HIT). HIT is a serious immune-mediated condition that paradoxically leads to a prothrombotic state despite anticoagulant use. It is characterized by a significant reduction in platelet count and an increased tendency for blood clot formation. These thromboembolic events associated with HIT can result in considerable morbidity and mortality among affected patients. Τhe estimated incidence of HIT is about one out of every 1500 hospital admissions annually and is associated with significant increase in patient morbidity, mortality, alongside with a general increase in the use of healthcare resources [32]. HΙΤ can arise from exposure to either unfractionated heparin (UFH) or low-molecular-weight heparin (LMWH). However, it usually and more frequently occurs with UFH due to the more intense immune response [33]. Around half of the patients affected by HIT experience thromboembolic complications, which may result in a mortality rate reaching as high as 30% [34,35].

Heparin-Induced Thrombocytopenia (HIT) is a strongly prothrombotic immune-mediated disorder that develops in response to the formation of pathogenic antibodies directed against complexes of platelet factor 4 (PF4) and heparin. Platelet factor 4 (PF4) is a positively charged protein stored in the alpha granules of platelets. Following heparin administration, PF4 is released and binds to the negatively charged heparin molecules, forming PF4–heparin complexes. These complexes expose new antigenic sites that are recognized as foreign by the immune system. Subsequently, IgG antibodies are generated against the PF4–heparin complex. The resulting immune complexes (PF4–heparin–IgG) bind to FcγIIa receptors on circulating platelets, leading to platelet activation [36]. Activated platelets then release additional PF4, along with other prothrombotic and pro-inflammatory mediators, further amplifying platelet activation and aggregation. This self-perpetuating cycle results in platelet consumption (thrombocytopenia) accompanied by a markedly increased risk of thrombosis, most often venous but occasionally arterial [35]. HIT immune-driven adverse reaction may develop within hours or a few days following heparin administration [37]. Several factors influence the likelihood of developing HIT, including the duration of heparin therapy, the type of heparin administered (with unfractionated heparin posing a greater risk than low-molecular-weight heparin, the dosage, the patient population, and gender, as women are more predisposed to HIT than men [38].

Heparin-Induced Thrombocytopenia (HIT) is classified into two types. Type I HIT is the more common form, occurring in about 10–30% of patients, typically within the first 48–72 h after exposure to heparin. It is characterized by a mild, transient decrease in platelet count and is considered a benign condition that does not lead to thrombosis. In contrast, Type II HIT is an immune-mediated reaction caused by antibodies directed against platelet factor 4 (PF4)–heparin complexes. This form usually develops 5–14 days after the initiation of heparin therapy (or sooner in previously exposed individuals) and is associated with significant thrombocytopenia and a high risk of thrombotic complications [39]. Thrombosis in HIT can be venous, arterial, or microvascular and may involve any organ [40].

### 2.5. HIT and Cardiac Surgery

The incidence of HIT is higher in patients undergoing cardiac surgery: 1.1%, and with ECMO use may reach up to 4.2% [41,42]. For cardiopulmonary bypass (CPB), heparin is administered at an initial dose of 300–400 IU per kilogram of total body weight, aiming to maintain an activated clotting time (ACT) within a range of 400–480 s [43].

In cardiac surgery, there are several mechanisms that can lead to a decrease in platelet count. Thus, thrombocytopenia may occur due to the use of intravascular devices or cardiopulmonary bypass (CPB). The use of an intra-aortic balloon pump (IABP) can reduce platelet count by up to 40% [44]. The most common pattern observed in cardiac surgery patients is the ‘biphasic fall,’ in which platelet counts initially recover 48–72 h after the first drop, followed by a second decline occurring 5 to 10 days after heparin exposure [45].

### 2.6. Diagnostic Evaluation of Thrombocytopenia and HIT Suspicion

Initially, pseudothrombocytopenia should be excluded, as it represents a laboratory artifact. This condition results from in vitro platelet clumping in blood collection tubes containing the anticoagulant EDTA, leading to a falsely low platelet count in automated measurements. To confirm or rule out this possibility, the platelet count should be repeated using a citrate-containing tube instead of EDTA. In addition, the use of glycoprotein IIb/IIIa inhibitors may cause both true and spurious thrombocytopenia, and this should be considered when interpreting the results [46]. The basic laboratory evaluation for thrombocytopenia should begin with a repeat platelet count in a citrated tube to exclude pseudothrombocytopenia. A coagulation profile including INR, PTT, fibrinogen, and D-dimer should be performed to assess for potential disseminated intravascular coagulation (DIC) or other coagulopathies. Examination of the peripheral blood smear provides essential diagnostic clues: schistocytes indicate possible thrombotic microangiopathy, blasts may reveal acute leukemia, atypical lymphocytes can point to certain viral or atypical infections, and spherocytes may indicate autoimmune hemolytic anemia, as seen in Evan’s syndrome. If HIT is suspected in a patient receiving heparin who develops thrombocytopenia the 4Ts Score should be investigated [47] [Table 2].

The probability of heparin-induced thrombocytopenia (HIT) can be assessed using the 4Ts scoring system, which evaluates four clinical domains: the degree of thrombocytopenia, the timing of the platelet count fall in relation to heparin exposure, the presence of thrombosis or other related complications, and the likelihood of alternative causes for thrombocytopenia. Each parameter is assigned a score from 0 to 2 based on the strength of clinical evidence.

A high score (2 points) is given when the platelet count decreases by more than 50% with a nadir of at least 20 × 10^9^/L, when the onset of thrombocytopenia occurs typically between days 5 and 10 post heparin exposure or within one day if the patient was exposed to heparin in the previous 30 days, when new thrombosis, skin necrosis, or an acute systemic reaction after a heparin bolus is observed, and when no alternative explanation for thrombocytopenia is evident.

An intermediate score (1 point) applies when the platelet count falls by 30–50% or the nadir is between 10–19 × 10^9^/L, when the timing of onset is unclear (for example, due to missing platelet data), or it occurs after day 10, or within one day in cases of prior heparin exposure 30–100 days earlier, when thrombosis is suspected but stays unconfirmed or even when erythematous skin lesions appear, and lastly when an alternative cause for thrombocytopenia is possible but not definite.

A low score (0 points) is assigned when the platelet count falls by less than 30% or reaches a nadir below 10 × 10^9^/L, when the decrease occurs within four days without recent heparin exposure, when no thrombotic manifestations are present, and when a definite alternative cause explains the thrombocytopenia.

The total 4Ts score ranges from 0 to 8, categorizing patients into low (0–3), intermediate (4–5), or high (6–8) probability groups for HIT. In cases when there is a score of 4 or higher, confirmatory laboratory testing, such as anti-PF4/heparin antibody ELISA or serotonin release assay, is recommended to establish the diagnosis.

The management of heparin-induced thrombocytopenia (HIT) is guided by established hematology society recommendations. Major reference sources include the British Society for Haematology (BSH) consensus statements and the American Society of Hematology (ASH) guidelines [40,48].

When the 4Ts score is ≤3, the clinical probability of heparin-induced thrombocytopenia (HIT) is considered low, and further laboratory testing is generally not indicated.

When the 4Ts score is ≥4, the clinical probability of heparin-induced thrombocytopenia (HIT) is intermediate to high. In such cases, all forms of heparin, including low-molecular-weight heparin, should be discontinued immediately and, if anticoagulation is required, a non-heparin anticoagulant should be initiated.

Subsequently, an immunoassay for anti–platelet factor 4 (PF4)/heparin antibodies should be obtained. A negative immunoassay effectively excludes HIT, allowing heparin therapy to be resumed and non-heparin anticoagulation to be discontinued. However, if the immunoassay is positive, the patient should continue to avoid heparin while remaining on the alternative anticoagulant until a confirmatory functional assay, such as the serotonin release assay, is performed [49]. Immunological assays for HIT are laboratory tests designed to detect and measure antibodies directed against the heparin–platelet factor 4 (PF4) complex. Common methods include solid-phase ELISA, which binds PF4/heparin complexes to a surface and detects antibody binding through an enzymatic color reaction; liquid-phase ELISA, which allows antigen–antibody interaction in solution for a more physiological setting; and the gel particle immunoassay (ID-Diamed), which uses microgel particles to visually demonstrate antibody-induced agglutination [50]. Functional assays in HIT testing aim to assess whether the identified anti-PF4/heparin antibodies can actually trigger platelet activation, a process that can lead to thrombosis. In contrast to immunoassays, which only confirm the presence of antibodies, functional tests determine their capacity to produce a biological response. The serotonin release assay (SRA) serves as the reference method, as it quantifies serotonin released from donor platelets when exposed to a patient’s antibodies and heparin. Additional techniques, such as the heparin-induced platelet activation (HIPA) test and flow cytometry-based assays, evaluate platelet activation through aggregation or specific surface markers [51].

**Figure 2:**
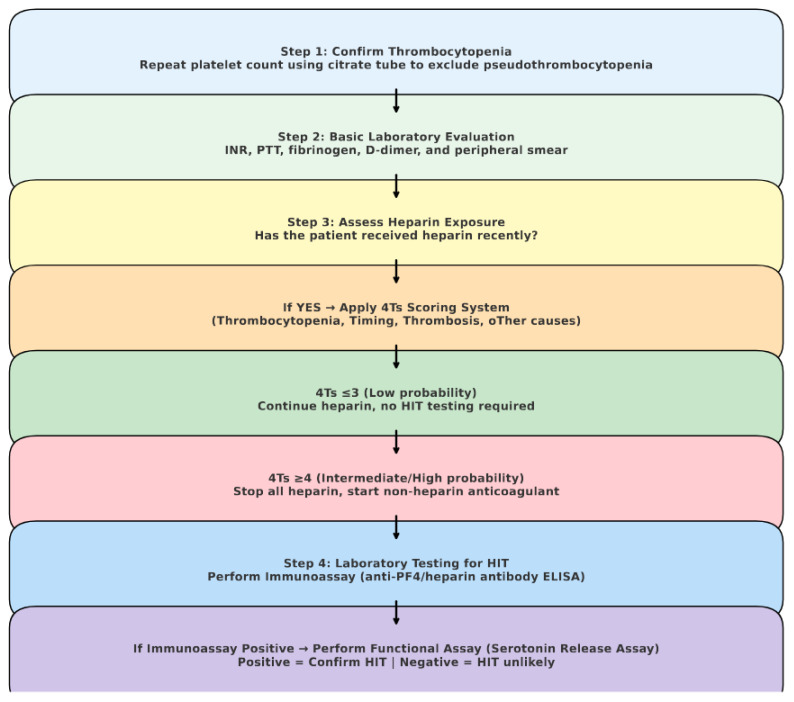
Diagnostic Algorithm: Thrombocytopenia and Heparin-Induced Thrombocytopenia. (HIT).

A positive functional assay confirms the diagnosis of HIT, whereas a negative result makes HIT unlikely and permits reassessment of the anticoagulation strategy. This algorithmic approach allows for safe and efficient management of patients with suspected HIT by integrating clinical probability scoring with laboratory confirmation [Figure 2].

Τhere are 5 phases of HIT: Suspected HIT, Acute HIT, Sub-acute HIT A (platelet count recovers but functional assay and Immunoassay are positive), Sub-acute HIT B (platelet count recovers, functional assay is negative and Immunoassay is positive), Remote HIT(platelet count recovers, functional assay and Immunoassay are negative).

Several diagnostic algorithms have been developed based on the clinical 4Ts score. Although the 4Ts score has an excellent negative predictive value (NPV), approaching 100%, its usefulness is limited by a relatively modest positive predictive value (PPV) and notable inter-observer variability. This has created an urgent need for improved diagnostic tools for heparin-induced thrombocytopenia (HIT). In response, the HIT Expert Probability (HEP) score was developed using the consensus opinions of 26 specialists in the field [52]. The HEP score is a more detailed assessment system, but prospective multicenter validation is still required. It evaluates several clinical and laboratory parameters, including the magnitude of platelet count decline, nadir platelet count, skin necrosis at heparin injection sites, acute systemic reactions following intravenous heparin bolus, presence of bleeding or extensive bruising, and potential alternative causes of thrombocytopenia such as chronic platelet disorders, newly initiated non-heparin medications, severe infection, disseminated intravascular coagulation (DIC), indwelling intra-arterial devices (e.g., VAD, IABP, ECMO), and recent cardiopulmonary bypass within 96 h. To date, no large-scale or prospective studies have validated the HEP score, and current evidence does not demonstrate superiority over the 4Ts score. Recent British Society for Haematology guidelines indicate that either the 4Ts score or the HEP score may be used in clinical practice [40]. Emerging data suggest that the HEP score may be reliable in surgical ICU patients, with a score < 5 potentially useful for ruling out HIT in this population.

The Lillo–Le Louet (LLL) score is a specialized risk-assessment tool designed to estimate the probability of heparin-induced thrombocytopenia (HIT) in patients following cardiopulmonary bypass (CPB) surgery [53]. First described in 2004, the LLL score was developed to address the unique diagnostic challenges that arise in the cardiac surgery population, where thrombocytopenia is common and often multifactorial. Unlike general clinical scoring systems, the LLL score incorporates variables specific to the postoperative course of cardiac operations, including the characteristic biphasic platelet count pattern after CPB, the potential for repeated heparin exposure, and alternative causes of thrombocytopenia inherent to the cardiac surgery setting. It is based on three independent risk factors: a biphasic platelet count decline, an interval of five or more days between CPB and suspected HIT, and a CPB duration of 118 min or longer. Studies have shown that the LLL score offers higher specificity in the post–cardiac surgery environment compared with broader tools, making it a valuable diagnostic approach in distinguishing HIT from more common CPB-related platelet changes. Table 3 illustrates the comparative characteristics and differences between the major HIT scoring systems

### 2.7. Management

After HIT diagnosis, all forms of heparin must be immediately discontinued to prevent further platelet activation and thrombosis. Even minimal exposure, such as through catheter flushes should be strictly avoided. The risk of thrombosis remains high, even after discontinuation of heparin, reaching approximately 50% within the following 30 days [43].

Because of the significant risk of developing venous and/or arterial thrombosis, it is essential to initiate a non-heparin anticoagulant at full therapeutic dose. Non-heparin anticoagulants, although essential for preventing thrombotic complications, are associated with a higher risk of bleeding such as argatroban and bivalirudin which are indicated as treatments for acute HIT [54,55]. Danaparoid, fondaparinux, direct oral anticoagulant (DOAC) are also non-heparin anticoagulants.

Warfarin should not be used during the acute phase of HIT, as it can worsen the thrombotic risk causing a rapid decline in the natural anticoagulants protein C and protein S and leading to a hypercoagulable state [56]. For patients under warfarin therapy, reversal with vitamin K is suggested during the acute phase [57].

Parenteral non-heparin anticoagulants represent the first choice of therapy in acute HIT. Both bivalirudin and argatroban are direct thrombin inhibitors (DTI) and are administered as a continuous IV infusion.

As a general guideline, cardiac surgery procedure should be postponed in patients with history of HIT until the HIT antibodies are no longer detectable. This typically occurs more than 100 days after the initial diagnosis [40].

In cases of urgent surgical procedures, and high risk of bleeding argatroban or bivalirudin are often the preferred anticoagulants due to their shorter half-life and rapid reversibility. Such patients require careful and continuous monitoring. Individuals with moderate to severe hepatic impairment, argatroban should be avoided or administered at a reduced dosage. These parenteral anticoagulants are preferred in patients with HIT complicated by life- or limb-threatening thromboembolism (e.g., massive pulmonary embolism or venous limb gangrene).

According to the American Society of Hematology 2018 guidelines [47], there are the following stepwise recommendations for the practical management of HIT:

Upon suspicion of HIT (4Ts score ≥ 4), all forms of heparin must be discontinued immediately.

Therapeutic-dose anticoagulation with a non-heparin agent should be initiated without delay. Among the available options, argatroban and bivalirudin are recommended as first-line therapies, particularly for critically ill or perioperative patients, given their short half-lives and predictable pharmacokinetic profiles, allowing for safe use in hepatic or renal dysfunction. Fondaparinux or direct oral anticoagulants (DOACs) may serve as suitable alternatives in hemodynamically stable patients with preserved renal function and recovering platelet counts.

Warfarin should be avoided in the acute phase of HIT, as early initiation can precipitate venous limb gangrene by rapidly reducing natural anticoagulants protein C and S; it should only be started after platelet recovery to ≥150 × 10^9^/L, overlapping with the parenteral agent for at least five days.

For patients requiring cardiac surgery, the ASH panel advises postponing elective procedures until HIT antibodies are no longer detectable (typically >100 days after diagnosis). If surgery is urgent, bivalirudin is recommended for intraoperative anticoagulation, while heparin may be used only in combination with plasma exchange or potent antiplatelet agents (such as prostacyclin analogs or glycoprotein IIb/IIIa inhibitors).

These practical, phase-specific recommendations provide clinicians with a structured, evidence-based framework to minimize thrombosis and bleeding risks in both the intensive care and perioperative settings.

## 3. Conclusions

Thrombocytopenia is a common and clinically important finding among critically ill and cardiac surgery patients. Its causes are diverse, ranging from dilutional and consumptive mechanisms to immune-mediated destruction or reduced platelet production. Recognizing the underlying mechanism is essential for guiding appropriate management and avoiding unnecessary interventions.

In cardiac surgery, a transient postoperative decrease in platelet count is often expected, but a secondary decline or delayed recovery should always raise suspicion for heparin-induced thrombocytopenia or other pathological causes. Early identification using clinical scoring systems, supported by targeted laboratory tests, enables timely treatment and reduces complications.

A structured diagnostic approach, combined with awareness of procedure-related platelet changes, can significantly improve patient safety and outcomes in the perioperative and intensive care setting.

### Clinical Pearls

A secondary platelet fall after POD 5–10 is highly suggestive of HIT in cardiac surgery patients.

The 4Ts score reliably rules out HIT but lacks specificity after CPB.

The LLL score provides superior accuracy in post-CPB patients.

Device-related thrombocytopenia (IABP, Impella, ECMO, CRRT) commonly mimics HIT.

Immunoassays yield frequent false positives postoperatively; functional testing is essential for confirmation.

Heparin must be stopped immediately in intermediate/high-probability cases.

Argatroban or bivalirudin are preferred non-heparin anticoagulants in acute HIT.

Warfarin should not be started until platelet recovery.

Postoperatively, heparin should be avoided; fondaparinux or DOACs are safe alternatives.

## Figures and Tables

**Table 1:** Classification of Thrombocytopenia by Severity.

Severi	Platelet Count (/μL)	Clinical Notes
Mild	100,000–150,000	Usually asymptomatic
Moderate	50,000–100,000	No spontaneous bleeding, but increased bleeding risk with trauma
Severe	20,000–50,000	High risk of bleeding even with minimal trauma
Very Severe (Critical)	<10,000–20,000	High risk of spontaneous, life-threatening bleeding

**Table 2:** The 4Ts Score: Diagnostic Components and Interpretation.

Component	2 Points	1 Point	0 Points
Thrombocytopenia	Platelet fall > 50% and nadir ≥ 20,000	30–50% fall or nadir 10–19 k	Fall < 30% or nadir < 10 k
Timing	Clear onset days 5–10 or ≤1 day with recent exposure	Consistent with days 5–10 but not clear; or fall after day 10	Onset too early without exposure
Thrombosis	Proven thrombosis, skin necrosis, acute systemic reaction	Recurrent thrombosis, erythematous skin lesions	No thrombosis
Other causes	None apparent	Possible	Definite other cause

**Table 3:** Comparative characteristics and differences between HIT scoring systems.

Characteristic	4Ts Score	HEP Score	Lillo–Le Louet (LLL) Score
Purpose	General tool for estimating the probability of HIT	Enhanced general tool incorporating additional variables	Cardiac surgery—specific tool for HIT after cardiopulmonary bypass
Advantages	Excellent negative predictive value; easy to apply	Reduces some limitations of the 4Ts; incorporates additional factors	High specificity in cardiac surgery patients; tailored to CPB physiology
Limitations	Low positive predictive value; high inter-observer variability; often false-positive after CPB	Does not outperform the 4Ts in cardiac surgery; weaker in patients on RRT or with arterial devices	Requires greater familiarity; less widely used internationally
Suitability in cardiac surgery	Moderate—useful mainly for ruling out HIT	Moderate—not superior to 4Ts in cardiac surgery settings	High—specifically designed for cardiac surgery patients
Clinical application	Rule-out tool for HIT	Complementary assessment tool	Preferred (?) score in post-CPB patients

## Data Availability

Data available on request from the authors.
